# Profile and preliminary results of Iranian sub cohort chronic obstructive pulmonary disease (COPD) in Shahrekord PERSIAN cohort in southwest Iran

**DOI:** 10.1186/s12890-021-01469-8

**Published:** 2021-03-25

**Authors:** Fatemeh Zeynab Kiani, Ali Ahmadi, Akbar Soleymani Babadi, Hamid Rouhi

**Affiliations:** 1 Modeling in Health Research Center, Shahrekord University of Medical Sciences, Shahrekord, Iran; 2 Department of Epidemiology and Biostatistics, School of Health and Modeling in Health Research Center, Shahrekord University of Medical Sciences, Shahrekord, Iran; 3 Department of Internal Medicine and Pulmonary Disease, School of Medicine, Hajar Hospital, Shahrekord University of Medical Sciences, Shahrekord, Iran

**Keywords:** Chronic obstructive, Pulmonary disease, Longitudinal, PERSIAN Cohort study, Iran

## Abstract

**Background:**

Chronic obstructive pulmonary disease (COPD) is a chronic and complex respiratory disorder associated with airflow limitation and increased inflammatory response of the lungs to harmful particles. The purpose of this original study was to describe the results and profile of the Shahrekord Prospective Epidemiological Research Studies in IrAN (PERSIAN) regarding COPD in southwestern Iran.

**Methods:**

This study of asthma and respiratory diseases is a subcohort of the more extensive cohort study, i.e., Shahrekord PERSIAN cohort, a population-based prospective study on people aged 35–70 years in southwestern Iran (n = 10,075). The sample size of the subcohort was 8500 people. Annual follow-ups (person-year) of the cohort were designed to be conducted up to 2036. The instruments to collect data on various exposures were derived from the questionnaires previously developed in extensive multinational studies (occupational exposures, smoking, housing status, and fuel consumption, history of respiratory and chronic diseases, comorbidity, etc.). The Global Initiative for Chronic Obstructive Lung Disease (GOLD) and the lower limit of normal (LLN) spirometric criteria were used to confirm COPD diagnosis.

**Results:**

The response rate was 93.85%. The mean age of the participants was 49.48 ± 9.32; 47.9% were male, and 52.9% were female; nearly 16% of the population was current smokers; the fuel used by most of the participants for heating the house and cooking was gas. The most common comorbidity among participants was dyslipidemia; 30% of people have three or more comorbidities. According to GOLD and LLN criteria, the Prevalence of COPD was 3.6% and 8.4%, respectively. 4.3% of the participants had a history of chronic lung disease. The group of subjects with COPD had higher mean age, fewer years of schooling, a higher percentage of smokers with a smoking history of 10 or more pack years. 4.6% of patients had a history of chronic lung disease, 17.6% had a history of asthma in childhood, and 5.2% had a family history of respiratory and pulmonary diseases.

**Conclusion:**

Epidemiological research is necessary to create an appropriate framework to fight COPD. This framework requires a better description of men and women at risk of developing COPD and describing people with early-stage illnesses.

## Introduction

Chronic obstructive pulmonary disease (COPD) is a complex respiratory disorder that is caused by airflow limitation and increased inflammatory response of the lungs to harmful particles and gases, which is usually progressive and irreversible [1]. According to the World Health Organization, COPD is not a single disease but is a so-called umbrella disease that covers a wide range of pulmonary diseases, including emphysema and bronchitis [2]. It affects 6–10% of the world's population [3] and is one of the most important causes of mortality and disability across the globe [4]. The burden of COPD has risen over time [5], and the current costs associated with this disease are remarkable and will increase in the future [6, 7].

In a recently published meta-analysis study, the pooled Prevalence of COPD was 15.70%. Among all WHO regions, the highest prevalence was in the Americas, and the lowest was in the Southeast Asia/West Pacific region [8]. In Iran, the prevalence of COPD in estimating the burden of obstructive pulmonary disease was 8.3% [9]. The Prevalence of COPD in Tehran, the capital of Iran, was 9.2% [10] and in Isfahan, )neighboring Chaharmahal and Bakhtiari Province( was 5.7% [11]. In another study in Iran from 5 different geographical areas in Iran (north, south, east and center), the overall prevalence of COPD was 4.9%, which is the highest province of Kerman (13.9%) and then Tehran 4.4%, Ahvaz was 3.8%, Mazandaran was 3.7%, and Mashhad was 2.8%, respectively [12].

Despite the significant impact of COPD on health and the economy, this chronic disease has not yet drawn enough attention from the public healthcare institutes and is not known among the general population. One possible reason for this significant problem is the lack of epidemiological data on the prevalence and risk factors of COPD in developing countries, especially in Iran. According to previous studies, the diagnosis of COPD is underreported in Iran [10].

Evidence and epidemiological data on the status and progression of COPD in Iran are minimal and contradictory; the methods for examining this disease in various studies are different, and there is a paucity of evidence about the natural history of the disease. On the other hand, inconsistencies in the information on the Prevalence of COPD, chronic bronchitis and asthma in the Iranian population may affect the decision made by health care system officials, policy-makers and insurance organizations. They may prevent them from taking adequate preventive and treatment measures to prevent potential severe effects and stupendous costs [13].

Therefore, this longitudinal study was conducted to investigate the need for longitudinal observational studies on COPD in Chaharmahal and Bakhtiari province, southwestern Iran. The province is geographically located at approximately 2,153 m above sea level and is known as the roof of Iran. The effect of altitude on other medical conditions, such as pulmonary hypertension and heart failure, has previously been reported, but the potential mechanisms proposed for the effect of altitude on the Prevalence of COPD are highly contradictory and controversial. In the PREPOCOL-PLATINO-BOLD-EPI-SCAN study, authors claimed that " known risk factors were less frequent at high altitude and high altitude had no significant influence in COPD prevalence" [14]. In contrast, the PREPOCOL study results in five Colombian cities and four geographically diverse in Peru showed that the Prevalence of COPD increased with increasing altitude [15, 16].

In Iran, especially Chaharmahal and Bakhtiari province, no cohort study has yet been conducted to investigate the problems of lack of diagnosis of COPD and the need for intervention. Therefore, this work is a futuristic cohort study with a 20-year follow-up period in Chaharmahal and Bakhtiari, southwestern Iran, making it possible to do cross-sectional and longitudinal data analysis.

## Materials and methods

**Fig. 1:**
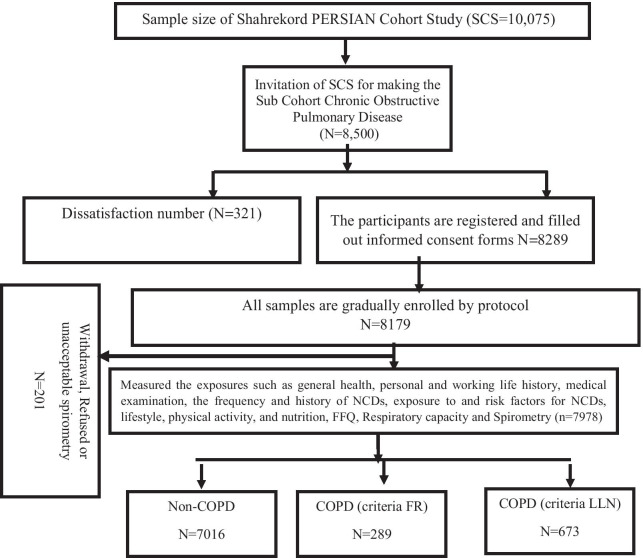
Summarizing Person's Recruitment and Assessment for Iranian Sub Cohort Chronic Obstructive Pulmonary Disease in Southwest of Iran

The study of asthma and respiratory diseases is a subcohort of a more extensive cohort study, i.e., Shahrekord Cohort Study (SCS), a population-based prospective study on people aged 35–70 years in southwestern Iran. SCS was designed to serve as one of the centers of the Prospective Epidemiological Research Studies in IrAN (PERSIAN) Cohort (n = 10,075) and is being conducted in the southwest of Iran [17]. The sample size of the subcohort was 8500 people. This study began in November 2015 in Shahrekord and has been scheduled to continue until 2036, with a total follow-up of 200,000 person-years each year (Fig. 1). Details of the protocol and the objectives of the Shahrekord PERSIAN cohort have already been published [18].

### Outcome definition

The primary outcome of this study is COPD, and mortality from COPD is an airway inflammatory disease. That is associated with continuous airflow limitation, which is usually progressive and irreversible. The most common symptoms of COPD are coughing, phlegm production and difficulty breathing, which should be considered for the clinical diagnosis of the disease [19]. However, none of the symptoms is sufficient to make a diagnosis, and if there are several additional symptoms and tests to diagnose the disease, the likelihood of a diagnosis of COPD increases. According to ICD-10, COPD includes emphysema and chronic bronchitis. Chronic coughing is usually the first symptom that occurs. Chronic bronchitis is defined as a condition of cough and sputum for at least three months for two consecutive years during the past year. Diagnosis of emphysema is only possible by describing the changes in the anatomy of the lung tissue and cannot be considered a disease per se [1, 20]. The most common and easiest way to confirm the diagnosis of COPD is spirometry. Most studies have only used a questionnaire to diagnose COPD, but in this study, both instruments were used for the diagnosis of COPD (Table1). Data collection methods and exposure variables in a subcohort of Shahrekord PERSIAN Cohort were reported in Table 1.

**Table 1:** Data collection methods, exposure variables and tools in a subcohort of Shahrekord PERSIAN cohort

Data and collection methods	Exposure variables
Questionnaires (self-reported)	History and current occupational exposures, personal history and habits (smoking, alcohol, tobacco smoking and drinking), as well as sedentary time, the age at which smoking began and the stages of change of readiness to quit smoking in current smokers, fuel status for home heating and cooking, Housing status, history of contact with animals, exposure to agricultural toxins, pesticides and detergents Medical history including pulmonary diseases, the history of asthma in childhood, respiratory symptoms, respiratory infections, chronic illnesses, drug use, family history of respiratory and pulmonary diseases, and questions about whether *Have you ever had a doctor or other health care professionals diagnose one of the following conditions: Chronic bronchitis, emphysema, pulmonary fibrosis and sleep apnea* Comorbidities: Cardiovascular disease (Myocardial Infarction, Cardiac Ischemia, Heart Failure, stroke), Hypertension, Type 2 diabetes, Metabolic syndrome, Dyslipidemia, Anxiety, Depression, Renal failure, Fatty liver, Musculoskeletal disorders, Pulmonary blood pressure, Gastroesophageal reflux disease (GERD), Pulmonary cancer, Pulmonary fibrosis
Clinical examination	Blood pressure, Heart rate, Electrocardiogram(EKG), Breathlessness on exertion
Anthropometry indexes	Height, Weight, Waist/Hip/Wrist Circumference, Body Components (fat, water, muscle), Bioimpedance
Blood samples	Fasting Blood Sugar, Serum Triglyceride, Total cholesterol, Low and High-Density Lipoprotein, Cholesterol
Spirometry	Pre- and Post-bronchodilator for diagnosis of COPD
COPD assessment test (CAT)	Only COPD and at-risk
Routine data and BioBank	Link all variables to Shahrekord PERSIAN cohort Data Base. A biobank that has been designed to store blood, nail, hair and urine samples for future research studies.

COPD, chronic obstructive pulmonary disease

A pulmonary function test was conducted by using a spirometer (New Spirolab, MIR, Italy, 2015) according to the criteria of the American Thoracic Society/European Respiratory Society (ATS/ERS). All tests were conducted in a quiet room in a sitting position on a comfortable chair. The spirometer was calibrated using a syringe by trained technicians daily before the study began. All participants were informed about all stages in the investigation and the pulmonary function test. All steps of the spirometry maneuver were performed practically by technicians so that the participant could see how to do proper inhaling and exhaling. The person was instructed to take a deep, full breath and then exhale forcefully. In-depth and complete inhalation is no less important than strong and complete exhalation. Inadequate and incomplete inhalation will lead to an insufficient volume of exhalation, resulting in a false decrease in forced vital capacity values and an increase in the likelihood of a restrictive pattern. Pulmonary function tests were conducted in triplicate for each individual with a single and acceptable method. By comparing the curves of the three pulmonary function tests, the maximal values of FEV1 (forced expiratory volume in one second) and FEV6 (forced expiratory volume in 6 second) forced vital capacity (FVC), maximum peak expiratory flow (PEF) in 25%, 50% and 75% of FVC (PEF25-75), Maximum Ventilatory Volume (MVV) were obtained. Spirometry data were interpreted according to the ATS/ERS recommendations by two respiratory medicine specialists. The pulmonary function test parameters values were presented as the percent of predicted values for the respective age, height, and weight [21].

The GOLD criteria (The Global Initiative for Chronic Obstructive Lung disease) uses a fixed ratio of forced expiratory volume in 1 second (FEV1) over forced vital capacity (FVC) < 0.7 for the diagnosis of COPD [22]. The post-bronchodilator spirometry was conducted for patients with a pre-bronchodilator FEV1/FVC ratio < 80% and 15 min after administration of 2 puffs (200 µg) of salbutamol via a spacer standard to evaluate the reversibility of the obstruction. We used a pre-bronchodilator FEV1/FVC ratio < 0·8 or an FVC < 80% as cutoffs for whether or not to do post-bronchodilator spirometry, to avoid underestimating FVC, which could result in a normal FEV1/FVC ratio. COPD was defined as the presence of post-bronchodilator FEV1/FVC of less than 70%. Although using this fixed ratio is easy and common, but the value varies greatly with age and decreases with age, thus leading to underestimation in adults under 45 years and overestimation in older people [23, 24]. For these reasons, the American Thoracic Society (ATS) and the European Respiratory Society (ERS) recommends setting the cut-off to 5% of normal to avoid potential Misclassification [25, 26]. Therefore, in this study, Spirometry data were expressed in predicted percentage according to the lower limit of normal (LLN), FEV1/FVC ratio < LLN, and also according to the GOLD criteria with a constant ratio FEV1/FVC < 0.70 and FEV1 < 80%. In addition, COPD severity was determined for all participants according to the GOLD criteria as follows: Stage 0 (at risk); stage 1 (mild): FEV1/FVC < 70% and FEV1 ≥ 80%; stage 2 (moderate): FEV1/FVC < 70% and 50% ≤ FEV1 < 80%; stage 3 (extreme): FEV1/FVC < 70% and 30% ≤ FEV1 < 50%; and stage 4 (extremely severe): FEV1/FVC < 70% and FEV1 < 30% [1, 27].

Contraindications for the use of spirometry drugs included cardiac infarction, pulmonary embolism, diagnosed aneurysm, uncontrolled blood pressure over 140 mmHg, previous surgery on the eyes, ears, brain, abdomen and chest, liver, heart or kidney failure, cancer, and endocrine disorders).

### Definition of exposures

A questionnaire was used to collect information about various exposures. After obtaining informed consent, complete information about various exposures was collected by experienced interviewers through face-to-face interviews. The main Questionnaire used in this study was derived from valid questionnaires that had been used in multinational studies. The main exposures of COPD disease are specifically studied. The Questionnaire also addresses the history and current occupational exposures, individual history and habits (smoking, alcohol, tobacco smoking and drinking), as well as sedentary time, the age at which smoking began and the stages of change of readiness to quit smoking in current smokers, fuel status for home heating and cooking, housing situation, history of contact with animals, exposure to agricultural toxins, pesticides and detergents.

All participants were asked questions about medical history, including pulmonary diseases, the history of asthma in childhood, respiratory symptoms, respiratory infections, chronic illnesses, drug use, family history of respiratory and pulmonary diseases, and questions about whether *Have you ever had a doctor or other health care professionals diagnose one of the following conditions: Chronic bronchitis, emphysema, pulmonary fibrosis and sleep apnea*.

Comorbidities about which the subjects were asked questions due to their clinical significance in COPD, included cardiovascular disease (myocardial infarction, cardiac ischemia, and stroke), hypertension, type 2 diabetes, syndrome metabolic, dyslipidemia, anxiety, depression, Osteoporosis, fatty liver, Rheumatoid Arthritis, pulmonary fibrosis [21].

In terms of smoking, the participants were divided into three groups as follows: Non-smokers, i,e., the people who never or occasionally smoked (those who have not yet smoked or have smoked less than 100 cigarettes during their lifetime); current smokers, i.e., the people who smoke one or more cigarettes a day; and ex-smokers, i.e., the people who are not currently smokers but smoked regularly in the past). Current exposure to cigarette smoke or passive smoking was also considered to be smoking-positive given the smoking of other family members or colleagues and exposure to parental cigarette smoke in childhood. Other additional variables such as anthropometric measurements and laboratory variables, which have already been published in the SCS protocol, were also collected [18]. The list of exposures and variables of this cohort were reported in Table 1.

### Generalizability of cohort (external cohort credibility)

Subcohort COPD was designed for a sample of approximately 8500 people out of 10,075 individuals aged 35–70 years for 20 years in Chaharmahal and Bakhtiari province, southwest of Iran. It seems that since this cohort contains a balanced ratio of men and women and urban and rural populations, it is likely to represent the community.

## Results

The response rate was 93.85%. Based on the results, 7978 people participated in the subcohort COPD, 6388 (80.1%) were urban, and 1590 (19.9%) were rural. The mean age of the population enrolled in the study was 49.48 (the standard deviation (SD), 9.32) years. 47.9% were male and 52.9% were female; 94.3% were married; 48.6% were of the Ethnicity Fars, and 39.1% were of the Ethnicity Lur Bakhtiari; 14.3% of the participants were illiterate, and 34.7% of participants were in the 35‐44 age category. 51.6% of the participants were housewives/unemployed/retired, and 18.2% were employees (Table 2).

**Table 2:** Baseline characteristics of the subcohort, by area residence urban/rural

Variables	Total N = 7978	Urban N = 6388	Rural N = 1590
Age—mean ± SD (years)	49.48 ± 9.32	49.04 ± 9.20	51.25 ± 9.58
35–44 years	2772 (34.7%)	2326 (36.4%)	446 (28.1%)
45–54 years	2708 (33.9%)	2185 (34.2%)	523 (32.9%)
55–64 years	1914 (24%)	1460 (22.9%)	454 (28.6%)
≥ 65	584 (7.3%)	416 (6.5%)	167 (10.5%)
Sex			
Men	3824 (47.9%)	3198 (50.1%)	625 (39.3%)
Female	4154 (52.1%)	3189 (49.9%)	965 (60.7%)
Ethnicity			
Fars	3874 (48.6%)	3721 (58.3%)	152 (9.6%)
Lur Bakhtiari	3122 (39.1%)	1775 (27.8%)	1347 (84.7%)
Turk Qashqai	649 (8.1%)	619 (9.7%)	30 (1.9%)
Other	333 (4.2%)	272 (4.3%)	61 (3.8%)
Number of family members—mean ± SD	4.00 ± 1.29	3.89 ± 1.14	4.45 ± 1.69
Number of family members—n (%)			
1	84 (1.1%)	59 (0.9%)	25 (1.6%)
2	782 (10%)	617 (9.9%)	165 (10.5%)
3	1745 (22.3%)	1477 (23.6%)	267 (17%)
4 or more	5205 (66.6%)	4096 (65.5%)	1109 (70.8%)
Marital status			
Single	137 (1.7%)	112 (1.8%)	25 (1.6%)
Married	7520 (94.3%)	6074 (95.1%)	1445 (90.9%)
Widow and divorced	321 (4%)	201 (3.1%)	120 (7.5%)
Education (N years)—mean ± SD	8.95 ± 6.01	10.16 ± 5.69	4.13 ± 2.08
Illiterate	1142 (14.3%)	593 (9.3%)	549 (34.5%)
≤ 5 years	1689 (21.2%)	1120 (17.5%)	569 (35.8%)
6–8 years	891 (11.2%)	702 (11%)	189 (11.9%)
9–12 years	2059 (25.8%)	1855 (29%)	203 (12.8%)
> 12 years	2197 (27.5%)	2117 (33.1%)	80 (5%)
Occupational status			
Employee	1450 (18.2)	1389 (21.7%)	60 (3.8%)
Farmer/rancher/herder	293 (3.7)	122 (1.9%)	171 (10.8%)
Carpet weaver/tailor/weaver	311 (3.9)	239 (3.7%)	72 (4.5%)
Heavy car driver/mechanic and oil change worker	507 (6.4%)	448 (7%)	59 (3.7%)
Building contractor/worker and builder	470 (5.9%)	311 (4.9%)	159 (10%)
Housewife/unemployed/retired	4120 (51.6%)	3142 (49.2%)	978 (61.5%)
Others	827 (10.4%)	736 (11.5%)	91 (5.7%)

The mean body mass index of the participants was 27.70% (SD, 4.58); 44.9% of the population had overweight (25–30) and 27.4% had obesity (> 30). Nearly 16% of the population was current smokers, with a higher proportion of men than women (33.3% VS 0.4%). The fuel used by most of the participants for heating the house and cooking was gas (65.2%) and also, the type of kitchen or cooking area for most participants was an open kitchen inside the house (74.9%). The most common comorbidity among participants was dyslipidemia (71.9%) and later hypertension (27.1%). 31.7% of participants had at least one comorbid and 30% of people have three or more comorbidities (Table 3).

**Table 3:** Baseline behavioral and clinical characteristics of the subcohort, by gender and area residence

Characteristics	Total N = 7978	Men N = 3824	Women N = 4154	Urban N = 6388	Rural N = 1590
Weight status	74.16 ± 13.27	78.15 ± 13.26	70.52 ± 12.20	75.49 ± 12.95	68.89 ± 13.27
Body mass index (BMI)—mean (SD)	27.70 ± 4.58	26.62 ± 4.02	28.68 ± 4.83	27.87 ± 4.47	27.00 ± 4.93
Underweight (BMI < 20)—n (%)	259 (3.3%)	165 (4.4%)	94 (2.3%)	163 (2.6%)	96 (6%)
Healthy (20–25)—n (%)	1934 (24.4%)	1100 (29.1%)	834 (20.2%)	1447 (22.9%)	487 (30.7%)
Overweight (25–30)—n (%)	3550 (44.9%)	1813 (48%)	1737 (42%)	2948 (46.6%)	601 (37.9%)
Obesity (> 30)—n (%)	2171 (27.4%)	699 (18.5%)	1472 (35.6%)	1768 (27.9%)	403 (25.4%)
Smoking status					
Current smoker n (%) yes	1288 (16.1%)	1272 (33.3%)	16 (0.4%)	1078 (16.9%)	210 (13.2%)
Former smoker n (%) yes	612 (7.7%)	596 (15.6%)	16 (0.4%)	511 (8%)	101 (6.4%)
Never smoker n (%) yes	6078 (76.2%)	1956 (51.2%)	4122 (99.2%)	4798 (75.1%)	1279 (80.4%)
Pack-years					
1–10	974 (12.2%)	777 (20.3%)	32 (0.8%)	728 (11.4%)	144 (9.1%)
10–20	583 (7.3%)	712 (18.6%)	0 (0.0%)	587 (9.2%)	100 (6.3%)
> 20	343 (4.3%)	379 (9.9%)	0 (0.0%)	274 (4.3%)	67 (4.2%)
Fuels used					
Gas	5198 (65.2%)	2519 (65.9%)	2679 (64.5%)	4777 (74.8%)	421 (26.5%)
Oil/gasoline	1801 (22.6%)	868 (22.7%)	933 (22.5%)	1183 (18.5%)	618 (38.9%)
Wood/firewood/animal dung	979 (12.3%)	437 (11.4%)	542 (13%)	427 (6.7%)	551 (34.7%)
Cooking area (kitchen type)					
Closed kitchen inside the house	1905 (23.9%)	826 (21.6%)	1079 (26%)	1371 (21.5%)	534 (33.6%)
Open kitchen inside the house	5979 (74.9%)	2951 (77.2%)	3028 (72.9%)	4951 (77.5%)	1028 (64.7%)
Outside of the house	94 (1.2%)	47 (1.2%)	47 (1.1%)	66 (1%)	28 (1.8%)
Kitchen ventilation status					
Ventilated	4422 (55.4%)	2277 (59.5%)	2145 (51.6%)	3899 (61%)	522 (32.8%)
Not ventilated	3556 (44.6%)	1547 (40.5%)	2009 (48.4%)	2488 (39%)	1068 (67.2%)
Comorbidities diseases status					
Cardiovascular disease	523 (6.6%)	306 (8%)	2.7 (5.2%)	429 (6.7%)	94 (5.9%)
Chronic lung diseases (asthma, tuberculosis, emphysema and bronchitis)	346 (4.3%)	155 (4.1%)	191 (4.6%)	302 (4.7%)	44 (2.8%)
Hypertension	2125 (27.1%)	1032 (27.6%)	1093 (26.7%)	1753 (28%)	372 (23.6%)
Diabetes mellitus	955 (12%)	424 (11.5%)	531 (13.1%)	815 (13.2%)	140 (9.2%)
Dyslipidemia	5610 (71.9%)	2579 (69.3%)	3031 (74.4%)	4451 (70.9%)	1159 (76%)
Metabolic syndrome	2006 (25.2%)	1660 (30.5%)	846 (20.4%)	1798 (28.3%)	208 (13.1%)
Anxiety and depression	1346 (16.9%)	353 (9.2%)	993 (23.9%)	1147 (18%)	199 (12.5%)
Musculoskeletal disorders	3929 (56.5%)	1562 (47.4%)	2367 (64.7%)	3316 (59.7%)	613 (43.8%)
Rheumatoid arthritis	385 (4.8%)	115 (3%)	270 (6.5%)	318 (5%)	67 (4.2%)
Osteoporosis	733 (9.2%)	59 (1.5%)	674 (16.2%)	615 (9.6%)	118 (7.4%)
Fatty liver	1280 (16%)	489 (12.8%)	791 (19%)	1134 (17.8%)	146 (9.2%)
Comorbidities—n (%)					
None	1211 (15.2%)	676 (17.7%)	535 (12.9%)	972 (15.2%)	239 (15%)
1	2529 (31.7%)	1265 (33.1%)	1264 (30.4%)	1848 (28.9%)	681 (42.8%)
2	1848 (23.2%)	849 (22.2%)	999 (24%)	1515 (23.7%)	333 (20.9%)
3 or more	2390 (30%)	1034 (27%)	1356 (32.6%)	2053 (32.1%)	337 (21.2%)

According to the GOLD criteria, 289 (3.6%) patients had COPD, and according to the LLN criteria, 673 (8.4%) had COPD. 4.3% of the participants had a history of chronic lung disease (asthma, tuberculosis, emphysema, and bronchitis), 11.9% had a history of asthma in childhood and 3.9% had a family history of respiratory and pulmonary diseases and also 13% of the participants had a history of chronic cough and of those who had a chronic cough, 47.6% had a history of chronic phlegm; 2.7% of the participants had Shortness of breath and wheezing. 47.8% of patients in the mild stage (GOLD I), 40.1% in the moderate stage (GOLD II), 9.8% in the severe stage of the disease (GOLD III) and 2.4% in the very severe stage of the disease (GOLD IV) were located (Table 4).

**Table 4:** Baseline airway obstruction in the subcohort of COPD and comparison between sex and residence group

Variables	All N = 7978	Men N = 3824	Women N = 4154	*P* value	Urban N = 6388	Rural N = 1590	*P* value
Airway obstruction—LLN	673 (8.4%)	345 (9%)	328 (7.9%)	0.039	558 (8.7%)	115 (7.2%)	0.029
Airways obstruction—FR	289 (3.6%)	159 (4.2%)	130 (3.1%)	0.008	243 (3.8%)	46 (2.9%)	0.045
Chronic lung disease (asthma, tuberculosis, emphysema and bronchitis)—n (%)	346 (4.3%)	155 (4.1%)	191 (4.6%)	0.127	302 (4.7%)	44 (2.8%)	< 0.0001
History of asthma in childhood—n (%)	949 (11.9%)	511 (13.4%)	438 (10.5%)	0.028	756 (11.8%)	193 (12.1%)	0.098
family history of respiratory and pulmonary diseases—n (%)	311 (3.9%)	138 (3.6%)	173 (4.2%)	0.261	251 (3.9%)	60 (3.8%)	0.879
Symptoms							
Chronic cough—n (%)	1038 (13%)	383 (10%)	655 (15.8%)	< 0.0001	937 (14.7%)	101 (6.4%)	< 0.0001
Chronic cough with phlegm—n (%)	494 (47.6%)	201 (52.5%)	293 (44.7%)	0.009	444 (47.4%)	50 (49.5%)	0.382
Shortness of breath and Wheezing—n (%)	217 (2.7%)	75 (2%)	142 (3.4%)	< 0.0001	188 (2.9%)	29 (1.8%)	0.007
Pre-bronchodilator spirometry (n = 7978)							
FEV1 (L.)—mean ± SD	2.84 ± 0.79	2.93 ± 0.81	2.76 ± 0.76	< 0.0001	2.92 ± 0.78	2.55 ± 0.74	< 0.0001
FEV6 (L.)—mean ± SD	2.08 ± 0.86	3.17 ± 0.87	2.99 ± 0.084	< 0.0001	3.16 ± 0.85	2.74 ± 0.80	< 0.0001
FVC (L)—mean ± SD	3.08 ± 0.86	3.18 ± 0.88	3.00 ± 0.84	< 0.0001	3.17 ± 0.85	2.74 ± 0.80	< 0.0001
FEV1/FVC—mean ± SD	92.48 ± 7.47	92.43 ± 7.63	92.52 ± 7.32	0.601	92.27 ± 7.45	93.33 ± 7.50	< 0.0001
FEV1/FEV6—mean ± SD	92.39 ± 7.48	92.60 ± 7.45	92.62 ± 7.32	0.678	92.42 ± 7.36	93.38 ± 7.41	< 0.0001
PEF (L)—mean ± SD	5.07 ± 2.09	5.28 ± 2.19	4.88 ± 1.96	< 0.0001	5.24 ± 2.12	4.37 ± 1.79	< 0.0001
MVV (L/min)—mean ± SD	99.67 ± 27.7	102.54 ± 28.3	97.02 ± 26.96	< 0.0001	102.27 ± 27.55	89.37 ± 26.21	< 0.0001
Post-bronchodilator spirometry (n = 761)							
FEV1 (L.)—mean ± SD	3.23 ± 0.21	3.41 ± 0.74	2.98 ± 0.88	< 0.0001	3.35 ± 0.42	2.78 ± 0.81	< 0.0001
FEV6 (L.)—mean ± SD	2.51 ± 0.74	3.67 ± 0.84	3.12 ± 0.36	< 0.0001	3.41 ± 0.65	2.98 ± 0.63	< 0.0001
FVC (L)—mean ± SD	3.89 ± 0.48	3.91 ± 0.73	3.84 ± 0.61	< 0.0001	3.99 ± 0.53	2.93 ± 0.61	< 0.0001
FEV1/FVC—mean ± SD	91.28 ± 6.94	91.40 ± 6.86	91.62 ± 6.82	0.687	91.39 ± 6.87	92.64 ± 6.91	< 0.0001
FEV1/FEV6—mean ± SD	92.73 ± 7.21	92.92 ± 7.69	92.89 ± 7.41	0.964	92.68 ± 7.44	93.81 ± 7.48	< 0.0001
PEF (L)—mean ± SD	5.51 ± 2.32	5.68 ± 2.38	5.21 ± 2.01	< 0.0001	5.84 ± 2.39	4.98 ± 1.94	< 0.0001
MVV (L/min)—mean ± SD	99.87 ± 28.1	102.66 ± 28.2	98.12 ± 28.91	< 0.0001	102.69 ± 27.97	90.21 ± 27.31	< 0.0001
Severity of COPD							
Mild	138 (47.8%)	92 (55.1%)	46 (37.7%)	0.003	111 (46.8%)	27 (51.9%)	0.801
Moderate	116 (40.1%)	52 (31.1%)	64 (52.5%)		98 (41.4%)	18 (34.6%)	
Severe	28 (9.8%)	19 (11.4%)	9 (7.4%)		22 (9.3%)	6 (11.5%)	
Very severe	7 (2.4%)	4 (2.4%)	3 (2.5%)		6 (2.5%)	1 (1.9%)	

LLN, low limit normal; FR, fixed ratio; FEV1, forced expiratory volume in 1 second; FEV6, forced expiratory volume in 6 second; FVC, forced vital capacity; PEF, peak expiratory flow

The group of subjects with COPD had higher mean age, fewer years of schooling, a higher percentage of subjects of smokers with a smoking history of 10 or more pack years. The fuel used by most of the participants for heating the house and cooking was gas (50.9%), and also, the type of kitchen or cooking area for most participants was an open kitchen inside the house (68.9%). 4.6% of patients had a history of chronic lung disease, 17.6% had a history of asthma in childhood and 5.2% had a family history of respiratory and pulmonary diseases (Table 5).

**Table 5:** Baseline behavioral and clinical characteristics of obstructed patients

Indicator	Fixed ratio		LLN	
	COPD (N = 289)	Non-COPD (6746)	COPD (N = 673)	Non-COPD (6547)
Weight status, mean (SD) kg	74.17 ± 14.18	74.16 ± 13.24	74.26 ± 13.19	74.15 ± 13.28
Body mass index (BMI)				
Mean (SD)	27.24 ± 4.52	27.71 ± 4.58	27.57 ± 4.56	27.71 ± 4.58
Underweight (BMI < 20)—n (%)	74 (25.7%)	246 (3.2%)	24 (3.6%)	235 (3.2%)
Healthy (20–25)—n (%)	130 (45.1%)	1860 (24.4%)	166 (24.9%)	1768 (24.4%)
Overweight (25–30)—n (%)	71 (24.7%)	3420 (44.8%)	299 (44.8%)	3251 (44.9%)
Obesity (> 30)—n (%)	75 (26%)	2100 (27.5%)	178 (26.7%)	1993 (27.5%)
Smoking status				
Current smoker	75 (26%)	1213 (15.8%)	159 (23.6%)	1129 (15.5%)
Ex-smoker	30 (10.4%)	582 (7.6%)	56 (8.3%)	556 (7.6%)
Never smoker	184 (63.7%)	5894 (76.7%)	458 (68.1%)	5620 (76.9%)
Pack-years				
1–10	51 (17.6%)	1171 (15.2%)	107 (15.9%)	1043 (14.3%)
10–20	36 (12.4%)	377 (4.9%)	71 (10.5%)	372 (5.1%)
> 20	18 (6.2%)	247 (3.2%)	37 (5.5%)	270 (3.7%)
Occupational status				
Housewife/unemployed/retired	133 (46%)	3972 (51.7%)	337 (50.1%)	3768 (51.6%)
Employee	47 (16.3%)	1403 (18.2%)	119 (17.7%)	1331 (18.2%)
Farmer/rancher/herder	21 (7.3%)	277 (3.6%)	33 (4.9%)	265 (3.6%)
Carpet weaver/tailor/weaver	12 (4.2%)	301 (3.9%)	25 (3.7%)	288 (3.9%)
Car driver/mechanic and oil change worker	20 (6.9%)	493 (6.4%)	41 (6.1%)	472 (6.5%)
Building contractor/worker and builder	24 (8.3%)	448 (5.8%)	45 (6.7%)	427 (5.8%)
Others	32 (11.1%)	795 (10.3%)	73 (10.8%)	754 (10.3%)
Fuels used				
Gas	147 (50.9%)	5051 (65.7%)	410 (60.9%)	4788 (65.5%)
Oil/gasoline	88 (30.4%)	1713 (22.3%)	164 (24.4%)	1637 (22.4%)
Wood/firewood/animal dung	54 (18.7%)	925 (12%)	99 (14.7%)	880 (12%)
Cooking area (kitchen Type)				
Closed kitchen inside the house	82 (28.4%)	1823 (23.7%)	174 (25.9%)	1731 (23.7%)
Open kitchen inside the house	199 (68.9%)	5780 (75.2%)	483 (71.8%)	5496 (75.2%)
Outside of the house	8 (2.8%)	86 (1.1%)	16 (2.4%)	78 (1.1%)
Kitchen ventilation status				
Ventilated	149 (51.6%)	4273 (55.6%)	360 (53.5%)	4062 (55.6%)
Not ventilated	140 (48.4%)	3416 (44.4%)	313 (46.5%)	3243 (44.4%)
Symptoms				
Chronic cough	32 (11.1%)	1006 (14.9%)	79 (11.7%)	958 (14.6%)
Chronic cough with phlegm	14 (45.2%)	480 (47.7%)	31 (41.9%)	463 (48%)
Shortness of breath and Wheezing	15 (5.3%)	202 (2.7%)	27 (4.1%)	190 (2.6%)
Chronic lung disease (asthma, tuberculosis, emphysema and bronchitis)	13 (4.6%)	333 (4.4%)	27 (4.1%)	319 (4.4%)
History of asthma in childhood	51 (17.6%)	898 (13.3%)	109 (16.1%)	840 (12.8%)
family history of respiratory and pulmonary diseases	15 (5.2%)	296 (4.4%)	39 (5.7%)	272 (4.1%)

COPD, chronic obstructive pulmonary disease; LLN, Low limit normal

## Discussion

Results of baseline cohort and profile publication are one of the most important outcomes after the completion of enrollment in cohort studies. This is useful for researchers, helps develop research fields and is helpful for health care system planners.

COPD is a preventable and curable disease [28]. Prevention of this disease should be taken into account as with other non-communicable chronic diseases, such as cardiovascular disease and cancer. Epidemiological studies are necessary to create an appropriate framework for fighting COPD. This framework requires a better description of men and women at risk of developing COPD and a description of people with an early-stage illness. This framework should also provide a better understanding of the risk factors that can be changed through interventions[29]. Subcohort COPD is the first longitudinal prospective study for COPD with population sampling in Iran. This study seeks to provide a broad description of the characteristics of men and women with COPD and to identify other causes of airway obstruction that can lead to an outcome. This study provides a unique opportunity to advance and consult observations on people with mild and unknown illnesses. The inclusion of healthy people and patients in the study provided a new opportunity to describe and follow a subgroup of the COPD patients who had not already been diagnosed, while many of them may at risk or have mild COPD that had not previously been diagnosed by a doctor or other health care professionals [30]. Most national estimates of COPD prevalence rates have been usually based on the data derived from the patients' self-report questionnaires and without an objective measurement of pulmonary function using a spirometer [10]. This study provides a good opportunity to assess the incidence of COPD by mean of both GOLD and LLN criteria from spirometry data so that its results can be compared with multivariate models of people who have not previously been diagnosed with COPD and who have COPD on the basis of known risk factors; in fact, this study examined the problems due to lack of COPD diagnosis and the need for intervention, and provides an opportunity to address the question *Will undiagnosed COPD be clinically important*? COPD is associated with a high incidence rate of one or more diseases. Comorbidities such as cardiovascular disease, musculoskeletal disorders, and metabolic syndrome are common in patients with COPD and significantly affect the quality of life of the patients, prognosis, and survival [31–34]. Increasing knowledge about the prevalence and effects of comorbidities in COPD is essential to adopt better intervention strategies and revise primary health care guidelines. COPD prognostic indicators currently focus primarily on prediction of mortality risk; creating a large COPD cohort for primary care and evaluating a wide range of outcomes enable us to review existing prognostic indicators and, if necessary, to develop new and appropriate prognostic indicators to predict other outcomes such as exacerbation and recurrence of disease, hospital admissions, and hospitalization due to exacerbation of respiratory diseases in primary care to be used in primary health care [30]. This study is a valuable work concerning increasing the longitudinal information and identifying the prognostic factors for COPD and the contribution of each of these factors to the progression and development of the disease due to its similarity to other cohort studies conducted around the world in terms of methodology, data collection methods and many other distinctive features [29]. Subcohort COPD is a good platform for standard research with Access to a database (such as lifestyle information, records and occupational exposures, smoking status and exposure to cigarette smoke and exposure time, fuel status, medical records, illnesses, and outcomes). Reported by patients, housing status and lung function test information, etc.). Using linear regression models with FEV1 as a dependent variable, we can estimate the progression of COPD over time. The data of this cohort study provide an appropriate infrastructure for the development of mathematical and statistical models to predict COPD and the survival rate of patients, and also the grounds for the analysis of the effects of various exposures, smoking and age using regression models and mortality rate from COPD as a dependent variable. There is a need for research to identify the impact of occupational exposure in COPD, especially among non-smokers by large, prospective and longitudinal studies. Therefore, in addition to the impact of occupational exposure, the potential interaction between occupational exposure and smoking was also addressed in this study. This study also provides a basis for answering an important and challenging research question about disease improvement and management, as well as interdisciplinary collaboration ranging from epidemiology to basic clinical research.

## Conclusion

We expect results from this and future research to help improve the health status and determine specific biological pathways or treatments for health care services planning and management decisions. Ultimately, this information will help policy-makers and public health decision-makers develop policies to improve the diagnosis, management and control of COPD. A biobank that has been designed to store blood, nail, hair and urine samples for future research is another strength of this study. Researchers who are interested in using the information can refer to the following web page: http://persiancohort.com.

## Data Availability

The study is ongoing. The general information is available from http://cohort.skums.ac.ir. All researchers across Iran and the world can have free access to the findings of this study, and necessary processes are available at the Cohort website to reproduce the research project, participate in collaborative research projects, and use the data. After requested, under conditions of collaboration and endowment, Access to the data is available for interested researchers from the corresponding author in A.A (aliahmadi2007@gmail.com).
